# Social Theory in Nursing Scholarship, From Humanism to Post‐Humanism: Revisiting S. Nairn on the Structure–Agency Debate

**DOI:** 10.1111/nup.70040

**Published:** 2025-09-22

**Authors:** Crystal A. West, Olga Petrovskaya

**Affiliations:** ^1^ School of Nursing University of Victoria Victoria British Columbia Canada

**Keywords:** agency, humanism, nursing, posthumanism, social structure, social theory, structure‐agency debate

## Abstract

Agency and structure are notable concepts in nursing philosophy and research. In this paper, we revisit two articles by Stuart Nairn which contrast selected theoretical perspectives exemplifying different positions within the structure‐agency debate. Nairn's chief argument in 2009 was that nursing scholarship overemphasized the role of agency while neglecting the concept of social structure. The goal of Nairn's articles was to remedy this asymmetrical perspective. Building on his analyses of Lévi–Strauss, Parsons, Giddens, Archer, and Bourdieu, and the nursing examples he employed, we critique and extend Nairn's contributions by situating the structure‐agency debate within the broader context of sociological theory. We suggest that his included theoretical perspectives largely assume agency to be an exclusively human attribute. Recent developments in critical posthumanism challenge this anthropocentric view, conceptualizing agency as distributed across human and nonhuman actors. Recognizing that differing approaches to structure and agency carry distinct implications for nursing knowledge and practice, we call for nurse scholars to make explicit their theoretical commitments when engaging these concepts in research and theorizing.

1

The concepts of *agency* and *structure* occupy a notable place in nursing philosophical and research scholarship. For example, analyses and critiques of social structures are evident in nursing's attention to the social determinants of health affecting patients and clients such as poverty, barriers to access education, and marginalized status due to demographic factors (Jackson et al. 2023; Johnson et al. 2022; Lathrop 2013; Northwood et al. 2018; Phillips et al. 2020; Schneiderman and Olshansky 2021). Other examples include nursing scholarship advancing social justice to recognize and address structural vulnerability of marginalized patient groups (Baah et al. 2019; Browne and Reimer‐Kirkham 2014; Fisher et al. 2024; Varcoe et al. 2014). In turn, the concept of agency in nursing scholarship is, for example, invoked in writings which call for emancipatory practice and resistance (Dillard‐Wright et al. 2023; Elliott 2024; Perron 2013, 2023; Thorne 2014).

Writing about agency and structure, nurse authors cite a range of theoretical perspectives (e.g., critical and feminist theory, postcolonial theory such as Bhabha's theory of hybridization, post‐humanist theory such as Latour's actor network theory and Bennett's agency of assemblages). However, López‐Deflory et al. (2023) integrative review of how the concept of agency is used in nursing research found that this concept is ‘not often explicitly defined’ (p. 3) and is frequently ‘detached from its theoretical or philosophical foundations’ (p. 4).

In this paper, we revisit and build upon two articles by Stuart Nairn, published in the Nursing Philosophy journal: *Social Structure and Nursing Research* (Nairn 2009) and *Pierre Bourdieu: Expanding the Scope of Nursing Research and Practice* (Nairn and Pinnock 2017). While Nairn foregrounded the concept of social structure (as evident from the title of his 2009 paper), his writing necessarily invoked agency as a foil to ‘structure’ in the structure–agency debate. These articles provide a useful overview and taxonomy of various theories explaining agency and structure.

Why are we undertaking this project? While applications of theoretical perspectives in original (primary) philosophical and empirical analyses are important, it is equally valuable to revisit quality summaries and overviews of the philosophers’ and social theorists’ work. Of particular value are summaries, like those by Nairn (2009), that contrast several related perspectives and differentiate among the usage of the ‘same’ concept by different theorists. In returning to the works of nurse author Stuart Nairn, this paper explores how agency is conceptualized across various social theories—specifically those of Lévi‐Strauss, Parsons, Giddens, Archer, and Bourdieu—each of whom viewed ‘human agency’ and ‘social structure’ as interrelated yet defined their nature and function in distinct ways.

## 2 Research Paradigms in Nursing Scholarship: A Brief History

To situate Nairn's arguments and the theories reviewed, it is useful to briefly trace the historical development of key philosophical and theoretical paradigms in nursing scholarship. It must be noted that not all nurse authors share terminology of ‘paradigms’—whether research or theory paradigms—and likewise, attempts to write ‘histories’ reflect the author's positionality within the field of nursing scholarship. In our usage, the word paradigm is synonymous with ‘waves’ or ‘developments’ and the story we relay is based on our reading of nursing literature including the sources we cite.

In the 1970–80s, to distinguish the discipline of nursing from biomedicine, nursing scholars advocated for a unique nursing knowledge, separate from positivist frameworks (Risjord 2010). Drawing from the philosophical foundations of Husserl's descriptive phenomenology, Heidegger's hermeneutic phenomenology, and Merleau‐Ponty's philosophy of embodiment, nurse scholars—such as Rosemarie Rizzo Parse, Jean Watson, Patricia Benner and Josephine Paterson with Loretta Zderad—brought interpretivist and phenomenological insights into American nursing theory and research (Paley 1997; Risjord 2010). These influences fostered a strongly humanistic orientation, emphasizing subjectivity, meaning‐making, and the nurse‐patient relationship as foundational to nursing.

From the late 1980s onward, nursing scholarship increasingly engaged with critical social theories and feminist perspectives, seeking to address the limitations of phenomenology's emphasis on the individual (Aranda 2016; Lipscomb 2016). Inspired by the work of Habermas, it remained critical of positivism and sought to liberate patients and nurses from systems of repression (Lipscomb 2016). (See Petrovskaya 2022a, pp. 22–23, for an overview of Habermasian nursing scholarship from the 1980s to early 2000s.) In the 1990s, the journal *Advances of Nursing Science* was a strong voice advocating a version of radical feminism protecting the nursing's ‘feminine’ qualities against male domination and medical patriarchy (Petrovskaya 2022a, pp. 85–86). Problematizing this assumption of women's universal experience, other nurse authors engaged with a wider feminist theory (Petrovskaya 2022a). For example, drawing on post‐structural feminism of Judith Butler and intersectional feminism of Patricia Hill Collins and Kimberlé Crenshaw, nursing scholars critiqued inequalities, power and privilege, and systemic oppressions shaping health and healthcare (Aranda 2016; Lipscomb 2016) while critically revisiting gender itself as being an effect of social performativity (Butler 1988) and positing a complex interaction of gender with other facets of human identity (Collins 1998). Further, several nurse researchers employed feminist epistemology of Dorothy Smith in their institutional ethnographies exposing hidden ruling relations impinging on nurses’ work of patient care. This critical and feminist shift reoriented nursing's focus from micro‐level practice to macro‐level structures. Within this framework, agency was understood as both individual and collective, rooted in conscious, human choice and resistance.

During the 1990s and 2000s, many nurse authors increasingly cited continental philosophy of post‐structuralism—most notably Michel Foucault's work skeptical of traditional views of both agency and structure. Yet, with some notable exceptions, nursing scholarship struggled to fully embrace the theoretical assumptions of Foucault's theoretical antihumanism and post‐structuralism (Petrovskaya 2022a). More recently, even nurse authors bidding ‘farewell to humanism’ (to use the expression from Petrovskaya 2023a) in favour of critical post‐humanism, arguably, do so without the intent to erase human agency per se. Currently, the diverse philosophical traditions outlined above, continue to inform and shape much of contemporary nursing research, theory, and literature.

## 3 Nairn's Overview of the Social Structure‐Agency Debate

### 3.1 Situating the Author and His Work

Stuart Nairn, a nurse researcher at the University of Nottingham, United Kingdom, is known for his contribution to the International Philosophy of Nursing Society (IPONS) including hosting the 18th annual IPONS conference in September 2014 (Nairn 2016a) in Nottingham. Nairn's publications in the *Nursing Philosophy* and other journals address the relevance of social theory and philosophy for nursing research and practice, adding to the body of knowledge informing nursing thought (e.g., Nairn 2012, 2016b, 2019).

In his article *Social Structure and Nursing Research*, Nairn (2009) examines a range of theoretical perspectives conceptualizing agency and social structure. Nairn contends that nursing qualitative research has largely foregrounded human agency while obscuring the role of social structures (p.192, 200). He explains this observation by two factors. First, nurses’ emphasis on the meaning of lived experience—of both patients and nurses—while relevant and understandable, has resulted in what Nairn describes as a ‘strongly humanistic philosophy’ (p. 194). Second, Nairn plausibly suggests that alternative sociological and anthropological theories analyzing social structure vis‐à‐vis agency might be unfamiliar (p. 195) or less likely to be taken up in some nursing quarters (p. 198). Nairn is careful not to diminish the importance of nursing studies which focus on the lived experiences of patients and nurses (p. 191, 200), such as the phenomenological studies he cites. At the same time, he argues that an overemphasis on agency over social structures limits our understanding of patients, as well as nurses’ experience and practice (p. 192, 200).

An important contribution of Nairn's (2009) paper is that he contrasts selected theoretical perspectives known in social sciences that exmplify the structure‐agency debate. Though Nairn does not provide an explicit definition of agency, consistent use of terminology throughout the article implies that agency is understood as active, individualistic, and humanist in nature, that is, attributed to and exercised by humans. According to Nairn (2009), social structure ‘refers to the patterns of behaviour that reoccur’ and provides the context in which behaviours transpire (p. 193). Nairn acknowledges that nuanced understandings of social structure vary among theoretical perspectives he reviews, as summarized below.

Nairn's taxonomy of structure‐agency theories includes four groups: (i) theories focused on agency, (ii) theories focused on structure, and theories addressing both agency and structure—including (iii) theories of duality (i.e., the reciprocal interdependence of agency and structure) and (iv) theories of dualism (i.e., the interaction of agency and structure as independent entities).

### 3.2 Theories Focused on Agency

The first group, theorizing that prioritizes human agency, is common to nursing research. These analyses, Nairn (2009) writes, tend to place responsibility on human action, erasing social structure. Social structures ‘exist through the constant acceptance [by an individual agent] of an interpretive interactional process’ of the social world; social structures are incorporated into individual actions and have no independent existence (p. 194). For example, the inability of nurses to provide holistic care, as in Nyström et al.'s study (2003 as cited by Nairn 2009), is attributed to a nurse's individual failing, rather than being recognized as a product of organizational imperatives of efficiency. Nairn lists phenomenology, hermeneutics, and interactionism as examples cited in the nursing qualitative research he reviewed (p. 192) but does not comment on whether it is those original philosophies or nurse researchers’ interpretation of them that result in a strong focus on individual human behaviour and agency. Other scholars have suggested that nursing's tendency toward discrepant interpretations of methodology and theory (Paley 1997, 2005; Petrovskaya 2023a; Risjord 2010) has contributed to this widespread humanist focus, which, in turn, overlooks the influence of structural, material, and nonhuman factors on nursing practice and knowledge development.

### 3.3 Theories Focused on Structure

The second group are theories which emphasize social structure and may be less familiar to nurse researchers. Nairn (2009) discusses two perspectives, Lévi‐Strauss’ anthropology and Parsons’ structural functionalism, and posits that a primacy of structure in these perspectives largely erases human agency and interaction from analysis (p. 197). According to the structural anthropology of Claude Lévi‐Strauss (1963, 1978), the social world rests upon immutable, universal truths which exist as rational yet unconscious mental operations in all of humanity. This natural, permeating truths manifest as behaviours, relationships and cultures. Structure then is the ‘invariant elements among superficial differences’ (Lévi‐Strauss 1978 as cited by Nairn 2009, p. 195). To illustrate this point, Nairn (2009) cites Lévi‐Strauss' (1963) work on kinship. Kinship structures seem highly variable at a first glance, but upon a structural analysis they share the central purpose of circulating women and organizing familial relationships. As such, these kin relationships express deeply rooted mechanisms (i.e., the incest taboo), as opposed to subjective, individual feelings of attraction (Lévi‐Strauss 1963 as cited by Nairn 2009).

While discussing the structural‐functionalist sociology of Talcott Parsons, Nairn focuses on the ‘sick role’ theory (Parsons 1951). In Nairn's interpretation of this study, individual behaviour is guided by social structures, namely norms, values, and expected roles. And behaviour has a function—to maintain the stability or development of the system, namely social structure and institutions. According to the sick role theory, individuals experiencing illness are socially obligated to make every effort to recover (e.g., seeking out medical advice and following treatment plans) and return to their normal role fulfillment (Parsons 1951). Doctors become gatekeeps determining who qualifies for the sick role, while patients become submissive subordinates. This view, Nairn argues, places an emphasis on the social structures governing the patient‐provider relationship, while ignoring the potential impact of the patient as autonomous consumer.

In his exposition of Parsons’ work, Nairn does not include Parsons’ more recent revisions of the sick role theory. We found that later work by Parsons (1975), Parson et al. (2001), Parson and Shils (2001) suggests a more reciprocal relationship between agency and structure. This is best described by Parsons and Shils (2001):

The structure of the social system…may be regarded as the cumulative and balanced resultant of many selections of many individuals, stabilized and reinforced by the institutionalization of value patterns, which legitimize commitment to certain directions of selection and mobilize sanctions in the support of the resultant orientations.

(p. 25)

Though Parsons (2007), Parson and Shils (2001) acknowledges that individual ‘selections’ contribute to shaping social structures, he maintains that action is predominantly controlled by social structure, supporting Nairn's classification.

## 4 Theories Accounting for Structure and Agency

Moving on from the first two categories in his taxonomy, Nairn (2009) turns to perspectives articulated by British sociologists that take both structure and agency into account. It is important, Nairn emphasizes, to differentiate between *duality* and *dualism*. Duality of structure, or the reciprocal interdependence of agency and structure, has been analysed by Anthony Giddens (1984) in his structuration theory. Dualism, or a view of agency and structure as ontologically separate but intertwined dynamically and temporally—Nairn's preferred view—has been articulated by Margaret Archer (1995). Additionally, we will review Pierre Bourdieu's theory of practice (Nairn and Pinnock 2017) in this part of the paper.

### 4.1 Theories Articulating Structure‐Agency Duality

According to the third category in Nairn's (2009) taxonomy, duality of structure, as articulated in Giddens' (1984) structuration theory, agency and structure are mutually constitutive, reciprocally shaping one another in simultaneity. This view contrasts functionalism and interactionism in that structure both produces and is produced by human agency. As per Giddens (1984), structure is the collective social rules and resources which shape behaviour, while agency is one's ability to act in contradiction to this influence (i.e., taking action which is not predetermined by structure). Human agents can modify or adapt their actions through the mechanisms of reflexivity, in response to structure, but their actions are constrained by these structures (Giddens 1984 as cited by Nairn 2009).

In an example provided by Nairn (2009), nurses may have the resources, skills and judgement to perform a certain procedure, but protocols and rules limit what they can and cannot do. Thus, social structure constrains action/agency but does not determine it. Nairn (2009) does not provide examples of duality from nursing research, except to highlight that according to Giddens’ concept of duality of structure, the nurse‐patient relationship, as a structure, only exists through social praxis. As structures are considered ontologically *virtual* until enacted by social agents, these relationships exist as *memory traces* until they are reproduced through social action (Giddens 1984 as cited by Nairn 2009). Therefore, these memory traces guide and shape nurse‐patient interactions, providing a pattern for these relationships (i.e., social structure), which can be analyzed through, and only through, their reproduction in nursing practice.

### 4.2 Theories Articulating Structure‐Agency Dualism

Nairn's (2009) last category in the taxonomy is illustrated by Archer's (1995) *analytical dualism*. Rooted in critical realism, this analytical method separates agency and structure, maintaining their distinct nature while allowing for their interdependence. Nairn (2009) explains that, whereas Giddens (1984) employs a methodological device of bracketing to isolate and analyze the inseparable entities of agency and structure, Archer (1995) posits not just a methodological differentiation but an ontological distinction of agency from structure to allow for independent analysis. As stated by Archer (1995), analytical dualism is predicated on two fundament concepts:

Firstly, it depends upon an ontological view of the social world as stratified, such that the emergent properties of structures and agents are irreducible to one another, meaning that in principle they are analytically separable. Secondly, it asserts that given structures and agents are also temporally distinguishable (in other words, it is justifiable and feasible to talk of pre‐existence and posteriority when dealing with specific instances of the two), and this can be used methodologically to examine the interplay between them and thus explain changes in both—over time.

(p. 66)

Structures are ontologically real human constructs which pre‐exist agency. Human agency is shaped by and responds to structure but is not reducible to structural influences on account of its independent causal power (Archer 1995 as cited in Nairn 2009).

Nairn (2009) illustrates a theory of structure‐agency dualism with an example of an interaction among a nurse, patient, and physician. In this relationship, each individual acts according to the roles, expectations and rules—the doctor diagnoses, the nurse implements treatments, and the patient complies with the treatment—structure within the interaction. Though this structure is rather constant (at least in a specific society, in a specific period), human agency allows for individuals to resist and reject structure thus resulting in contravening actions (e.g., the patient refuses to follow prescribed treatment) or deliberate removal from the context (e.g., a nurse may choose to work within an alternative institution).

Interesting, Nairn (2009) refers to a ‘collective agency’ within this taxonomic group. He argues that structural change can result from group agential action but is dependent on the position of the group within the structural relationships. For example, social change is more likely when put ‘forward by the government than say hospital porters’ (Nairn 2009, p. 199). To us, this suggests the interplay of hierarchy and power in collective action within social structures. It is Archer's analytical dualism that Nairn (2009) explicitly favours in his own approach to analysis stating it ‘reflects the ontological reality of the social world’ (p. 199).

Referencing Layder (1997), Nairn (2009) states this distinction between agency and structure suggests a stratified ontology of reality within a critical realist perspective. Parenthetically, we will note that Nairn did not elaborate on critical realism, except to ‘emphasize that social structure occupies a different domain from that of agency’ (p. 199). Further, though both Archer (1995) and Layder (1997) posit a stratified reality, we would point out that their understanding of the social layers of reality and their interplay is significantly different, but this is beyond the scope of this paper.

### 4.3 Additional Theories Pertaining to Structure and Agency

In a separate article, Nairn and Pinnock (2017) review the work of a French theorist, Pierre Bourdieu. Bourdieu's concepts of habitus, capital and field provide another point of access for nursing research to include both agency and structure in analysis. *Habitus*, ‘a system of dispositions,’ predispose an agent to do certain things, shaping an individual's future actions (Bourdieu 1977, p. 214). In Nairn and Pinnock's (2017) example, education and training develop the nurses’ professional habitus and affect how they will interact within the hierarchies of medical institutions. Therefore, they argue, habitus is structured and is itself a structuring structure.

*Field* is a structured social space where individuals and institutions interact (Bourdieu 1977 as cited in Nairn and Pinnock 2017). Each field (we can say, also integral to structure) has its own norms, rules, and power dynamics which influence individual action. Within the field of healthcare, nursing authority and action is defined by the policies and regulations which dictate what a nurse can and cannot do. Nairn and Pinnock (2017) explain the relation between field and habitus in the following way: ‘field could be described as the rules of the game whilst habitus is the feel for the game’ (p. 4).

Habitus and field are connected by various forms of *capital*—symbolic, economic, cultural and social (Nairn and Pinnock 2017). Capital refers to the resources such as knowledge, relationships, social position, prestige, or wealth which are used to gain power within a given field. The capital one possesses determines one's position and influence within a given field.

Nicolini's (2012 as cited in Nairn and Pinnock 2017) study of telemedicine in Italy exemplifies how Bourdieu's theory can aid researchers in analyzing nursing practice. With the introduction of the new telemedicine technology, nurses took on new skills and responsibilities, including the management of patient care plans, yet they continued to defer to physicians, demonstrating the internalization of nurses’ professional habitus (Nairn and Pinnock 2017). This example, we would point out, also highlights the hierarchical roles within the *field* of healthcare. The field delineates the rules and norms of nurses from those of physicians. Nairn and Pinnock (2017) suggest this example also highlights Bourdieu's element of capital. Though Nairn and Pinnock (2017) do not expand upon this connection, it could be said that the nurses’ increasing capital, through telemedicine training and newly acquired knowledge, could not outweigh the symbolic capital of doctors within the field of healthcare, resulting in the maintenance of hierarchical roles.

Nairn and Pinnock (2017) address criticism that Bourdieu's theory is deterministic in that it over‐emphasizes the influence of structure on action, presenting subjects as unreflective, thereby erasing human agency. To counter this, Nairn and Pinnock (2017) cite Bourdieu (1999) *The Weight of the World*. In his series of analyses of individual lives, Bourdieu (1999) locates individuals and their actions within the social. Nairn and Pinnock (2017) argue that this study is ‘full of reflective subjects’ (p. 5) highlighting Bourdieu's acknowledgement of deliberate individual choice and thus refuting critiques of determinism. We concur that Bourdieu's (1977, 1999) work does not negate agency in its attempt to explain the unconscious influence of structure on action; it positions *active* human agents within social realities through which deliberate, as well as unconscious, decisions and actions take place.

### 4.4 Theory as a Lens and an Intervention in the World

The choice of a theoretical perspective is not a neutral decision; it frames the questions which can be asked, the variables of focus, and the potential interpretation of findings. Theories intervene in the world and participate in the alternative world‐building (Law 2004; Mol and Mesman 1996). Differing theoretical perspectives offer distinct analytic advantages—capturing dynamic processes (e.g., Giddens), tracing structural change over time (e.g., Archer), revealing tacit logics and capital flows (e.g., Bourdieu), elucidating role stability (e.g., Parsons), or uncovering deeply rooted cultural codes (e.g., Lévi‐Strauss). At the same time, each carries limitations that, if unacknowledged, can constrain nursing scholarship's capacity to engage fully with the complexity of practice and its structural conditions. Table 1 provides a comparative overview of theories reviewed by Nairn and discussed above. Awareness of trade‐offs enable nurse scholars to make more deliberate, reflexive choices in their theoretical commitments. For example, Giddens’ structuration theory is advantageous for analyzing the day‐to‐day interactions and negotiations of nurses in practice as shaped by and shaping institutional norms. However, due to the analytical inseparability of structure and agency in Giddens’ duality, long‐term structural determinants of practice might become obscured. Bourdieu's theory of practice can be used to explore how nursing practice is governed by unarticulated norms and invisible logics, such as professional hierarchies. However, the lean toward determinism—through an overemphasis on structure—can limit the conceptualization of transformative agency. Ultimately, careful attention to the strengths and limitations of each theoretical approach enables nursing scholars to inform their research using theoretical assumptions and concepts which are best suited to illuminate their phenomena of interest.

**Table 1:** Comparison of structure‐agency theories addressed by Nairn.

Theorist	Main analytical focus	View of agency	View of social structure	Key assumptions	Advantages	Limitations
Levi‐Strauss	Structural Anthropology: universally deep structures shaping cultural practices	Agency is downplayed; human behavior is a product of unconscious symbolic structures	Structures are universal, binary systems which underly thought and culture	Meaning is derived from deep, unconscious codes	Illuminates underlying structures of human thought and culture and symbolic logics	Deterministic; neglects individual agency, context‐specific power and material conditions
Parsons	Structural Functionalism: social structures and roles maintaining system stability	Agency is constrained by social roles and norms; individuals conform to socially sanctioned behavior	Structures are systems of interrelated roles and norms that maintain social order	Society functions through value consensus; roles are functional necessities	Useful insight into enduring role expectations and institutional norms; how role expectations contribute to the functioning of systems; explain stability of certain practices	Overly normative; ignores power, resistance, and cultural diversity; critiqued for inadequacy in complex care contexts
Giddens	Duality of Structure: simultaneous co‐constitution of agency and structure	Agency is the knowledgeable, recursive conduct of actors who draw on structural rules and resources	Structures (rules & resources) are both the medium and outcome of social practices (*duality of structure*)	Structure and agency are inseparable in practice; structuration is recursive and ongoing	Allows for analysis of structure‐agency interactions in real‐time; applicable in empirical studies of professional routines and day‐to‐day practices	Difficult to analytically separate effects of agency from effects of structure, may obscure deeply embedded structures; limited attention to power and change; lacking temporal clarity
Archer	Analytical Dualism: ontological separation of structure and agency for temporal analysis	Agency is the capacity to reflect and act intentionally; agents are temporally distinct from structures and can transform them	Structures are pre‐existing, objectively real systems that condition but do not determine action	Grounded in critical realism; structure and agency are ontologically distinct and analytically separable; change happens via *morphogenesis*	Emphasizes temporal sequencing and reflexivity in dualistic relations; strong account of change; analytical distinction between structure and agency effects; useful for policy and systems change studies	Can be overly abstract; difficult to operationalize empirically; downplays continuous feedback of duality in lived experience
Bourdieu	Practice Theory: interplay of habitus, capital and field	Agency is shaped by *habitus;* agents take strategical action within the constraints of field and capital	Structures are relational fields (e.g. hospital hierarchies) and symbolic systems shaped by power and capital (economic, cultural, social).	Structure and agency are mutually constituted; *practices* arise from the interplay of habitus, capital, and field	Captures the influence of history and power on practice; exposes links between practice and broader social positioning; useful for analyzing inequality and professional norms	Risk of determinism if habitus is overemphasized; limited temporal analysis; transformative agency underexplored

### 4.5 Expanding Nairn's Work on Agency and Structure

In this section, we expand on Nairn's (2009), Nairn and Pinnock (2017) argument that differing conceptualizations of structure and agency have important implications for nursing research. Specifically, we discuss variations in classifications of some theories we reviewed, delineate *levels* of structure articulated by sociologists, and bring forward contrasting understandings of agency in humanist versus critical post‐humanist theory.

### 4.6 From Levels of Structure to Interconnections in Context

Our discussion of social structure throughout the paper is based on Nairn's (2009) definition of structure as ‘patterns of behaviour that reoccur’ (p. 134). This definition is widely accepted in sociology even when classifications of the structure‐agency theories slightly differ from that of Nairn's. For instance, Porpora (1989) outlines four conceptions of structure: (i) patterns of stable, recurrent behaviours; (ii) lawlike regularities which govern behaviour (e.g., Lévi‐Strauss); (iii) systems of human relationships related to social positioning (e.g., Bourdieu); and (iv) collective rules which structure behaviour (e.g., Giddens).

Critics could argue that understanding structure as ‘patterns of aggregate behaviour that are stable over time’ (Porpora 1989, p. 195) risks reducing analysis to individualist terms, thereby constraining explanations of macro‐level dynamics such as power and economics. Indeed, structure has been defined more broadly as the underlying framework of society that influences individuals and groups through the interrelations of institutions and social practices (Williams 2003). Another conception, familiar to nurses, demarcates levels of structure as follows: the micro level, encompassing interactions, interpersonal relationships, and self‐perception; the meso level, including groups and networks, including organizations, companies, and political parties; and the macro level, involving institutions (e.g., education, religion, health, gender) and systems (e.g., countries, governments, laws, economies) (Blackstone 2012; Juan and Requena 2006; Porpora 1996).

It is interesting to note a difference between social theorists’ understanding of structure as recurring patterns of behaviour (what can likely be placed at a micro level) and a characteristic use of the concept of structure in nursing literature as referring to macro‐factors—for example, capitalist, neoliberal societies reproducing systemic injustices. While nurses and sociologists (Crammond and Carey 2017; Øversveen 2023; Williams 2003) would agree that health is strongly shaped by the inequitable distribution of wealth, capacities and opportunities in society, the tendency in nursing research to utilize a wider, macro‐view of structure fails to recognize *social interactions* (e.g., as theorised by Giddens and Garfinkel's ethnomethodology) as a *constitutive element of structure* and as crucial for the development of nursing knowledge (Purkis 1994).

We suggest the consequences of viewing structures only as the so‐called macro‐entities are two‐fold: (a) imposing a fatalist view of health and patient experiences (Crammond and Carey 2017) and (b) negating the social construction of nursing practice at multiple levels (Purkis 1994). Likewise, understanding structures only as (micro) social interactions, patterned behaviours, and everyday exchanges, risks ignoring their interrelation with meso and macro factors. To overcome these limitations, Porpora (1996) suggests an approach which appreciates the likely appearance of structural forms at all levels and an awareness that ‘no inherent scope or locality intrinsically inheres in any given social form’ (p. 26). In a similar vein, Williams (2003) calls for ‘the need to theorise what people do as something more than either an individual lifestyle‐choice or the one‐way outcome of structural determinants which are themselves produced in some under theorised way by capitalism, post‐industrialism or globalism’ (p. 145). Indeed, nurse researchers should be careful to avoid forcing an analysis or theorization to fit an a priori structural limitation (Purkis 1994).

### 4.7 Agency in Humanist and Post‐Humanist Theory

Theoretical perspectives discussed throughout this paper ascribe agency exclusively to humans. Although structure shapes or co‐creates agency, it is still us, human beings, who are active, intentional, and enact change. Until the second half of the 20th century, this anthropocentric view was largely unchallenged in Western social theory. Consequently, as discussed above in a brief history of nursing research paradigms, nursing scholarship has predominantly drawn on humancentric social theory which posits agency as an inherently and uniquely human characteristic. However, in the last decade, nurse authors have increasingly engaged with a cluster of relatively novel strands of social theory where agency is conceptualized quite differently. These include material semiotics (Law 2023; Mol and Mesman 1996), actor network theory (Latour 2005), sociomaterial approaches from organizational studies (Orlikowski 2007), and critical post‐humanism and material feminism including Donna Haraway's (2016) cyborg ontology and Karen Barad's (2007) agential realism. These perspectives disrupt humanist assumptions by attributing agency not only to people but also to technologies, artefacts, infrastructures, and environments. From a socio‐material view, agency is not an inherent property of human actors but is an outcome (or effect) of relational networks among human and nonhuman actors. In other words, these theories not only extend ‘agency’ to nonhuman entities, but more radically, these perspectives revise the notion of agency itself—not as an inherent or a priori attribute, but a contingent effect (or outcome) of relations within a heterogeneous network. Thus, both humans and non‐humans may or may not be agential, depending on the relational configurations in which they are situated.

To take one example, in nursing research which examines clinical practice in healthcare settings that utilize electronic health records (EHR), these approaches have illuminated how EHR, monitoring devices, and other technologies actively shape care practices, professional roles, and patient outcomes (e.g., Booth et al. 2016; Petrovskaya 2023b). This reframing positions agency as distributed, emerging from the relational interplay of human and nonhuman entities, and responsive to both material and discursive conditions. In nursing, this is relevant for the analyses of technology's effects on care practices (Bignell and Petrovskaya 2024; Booth et al. 2016; Petrovskaya 2022b, 2023b) but is not limited to the field of human‐machine interaction. Posthumanism and material feminism in nursing scholarship, notably those engaging with Karen Barad's work (Aranda 2019; Smith et al. 2022), provide an important lens for examining inequality. From this view, inequalities are understood as sociomaterial products which emerge from heterogenous entanglements, suggesting new approaches to inclusive political action and ethical care.

## 5 Conclusion

In this paper, we revisited two articles by Stuart Nairn (2009), Nairn and Pinnock (2017) on concepts of structure and agency in sociology and nursing research. By illustrating theories of Lévi‐Strauss, Bourdieu, Archer, Giddens and Parsons with examples from nursing, Nairn demonstrates the implications of varying analyses of agency and structure on our understanding of nursing. Our critical commentary expanded Nairn's work. We juxtaposed various definitions of structure (i.e., a recurring pattern of behaviour, social systems, and levels) to underscore the multitude of perspectives which may shape, focus, or limit nursing knowledge. We noted predominantly humanistic assumptions underpinning conceptions of agency in reviewed theories and contrasted those with alternative conceptions of agency in post‐humanist theory.

Across historical shifts in nursing literature—from phenomenology and humanism, through critical and feminist theory, to post‐structural, post‐humanist and socio‐material perspectives—the concepts of ‘agency’ and ‘structure’ have been variably defined, reinterpreted, and, at times, inadequately addressed. To address this, we encourage nurse scholars to ground their analyses in clearly articulated theoretical perspectives, making explicit their conceptualizations of ‘agency’ and ‘structure’ in relation to a chosen philosophical orientation. This approach might sharpen theoretical analyses and also informs how agency is operationalized in research, policy, and practice. We also invite engagement with multiple theoretical lenses, recognizing that diversity can enrich and expand our understandings of nursing and the phenomena it seeks to explain.

## Ethics Statement

The authors have nothing to report. This study analyzed publicly available literature and did not involve human participants or personal data.

## Conflicts of Interest

The authors declare no conflicts of interest.

## Data Availability

Not applicable to this article as no datasets were generated or analysed during the current study.
